# Mechanisms of Thrombocytosis in Iron‐Deficiency Anemia

**DOI:** 10.1111/ejh.70211

**Published:** 2026-05-07

**Authors:** João Vitor Facco, Diane S. Krause, Sara T. Olalla Saad

**Affiliations:** ^1^ Hematology and Transfusion Medicine Center (Hemocentro) University of Campinas (UNICAMP) Campinas SP Brazil; ^2^ School of Medical Sciences University of Campinas (UNICAMP) Campinas SP Brazil; ^3^ Blood National Institute of Science and Technology (INCT Sangue) Campinas SP Brazil; ^4^ Department of Laboratory Medicine, Yale School of Medicine Yale University New Haven Connecticut USA; ^5^ Department of Cell Biology, Yale School of Medicine Yale University New Haven Connecticut USA; ^6^ Department of Pathology, Yale School of Medicine Yale University New Haven Connecticut USA; ^7^ Yale Stem Cell Center Yale University New Haven Connecticut USA

**Keywords:** hematopoiesis, iron‐deficiency anemia, iron metabolism, megakaryocytic‐erythroid progenitor, thrombocytosis

## Abstract

Iron‐deficiency anemia is frequently accompanied by reactive thrombocytosis, yet the mechanisms underlying this association remain incompletely understood. Beyond impaired erythropoiesis, iron availability has emerged as an active regulator of hematopoietic lineage decisions. This review synthesizes current evidence on how iron deficiency influences megakaryocytic–erythroid balance, with emphasis on progenitor‐level regulation, iron sensing pathways and downstream megakaryocyte maturation. We discuss experimental and clinical studies supporting compensatory hematopoietic responses under iron‐deficient conditions and propose an integrated framework linking systemic iron status to altered platelet and erythroid output.

## 1 Introduction

The pathophysiological mechanisms of the thrombocytosis that is observed in patients with iron‐deficiency (ID) anemia remain poorly understood [1, 2, 3, 4]. Iron‐deficiency, one of the most common causes of anemia worldwide [5], leads to reduced iron availability for erythropoiesis and decreased red blood cell production and results in symptoms such as fatigue and exercise intolerance [6, 7, 8]. Concordant with the decreased erythroid mass, 12%–28% of patients with ID anemia have an increased circulating platelet count [1, 2, 9, 10]. While in most cases thrombocytosis associated with ID anemia is asymptomatic, persistent elevation in platelet counts increases the risk of thrombotic events, particularly in vulnerable populations [2, 3, 11]. Furthermore, understanding the mechanisms underlying this phenomenon may shed light on novel interactions between the erythropoietic and megakaryocytic systems, contributing to a broader understanding of hematopoiesis and its regulation under conditions of nutritional deficiency. The literature presents several hypotheses to explain the increase in platelet count in iron deficiency, including changes in angiogenic factors such as VEGF (vascular endothelial growth factor) [12, 13, 14], transferrin receptors [12], hypoxia sensors such as HIF [13], and intracellular signaling pathways such as MAPK [15, 16]. The main proposed mechanisms linking iron deficiency to reactive thrombocytosis, together with the supporting experimental evidence, are summarized in Table 1. Each of these hypotheses offers plausible, yet isolated, mechanisms, and it is likely that they are interdependent.

**TABLE 1:** Mechanisms of thrombocytosis in iron deficiency.

Mechanism/pathway	Experimental model	Key findings and summary	Relevance to iron deficiency–related thrombocytosis	Author
VEGF/VEGFR1	In vitro: Primary human CD34+ HPCs	↑ VEGFR1 during MK differentiation VEGF‐VEGFR1 blockade ➔ ↓ Mk maturation. VEGF or VEGFR1‐specific ligand ➔ ↑ Mk maturation and polyploidization	Establishes VEGF‐VEGFR1 axis as regulator of MK maturation; indirect in ID.	Casella et al. (2003)
VEGF/VEGFR2	In vitro: TF1 cell line and primary human CD34+ HPCs	VEGFR2 activation ➔ ↑ MK commitment and maturation Context‐dependent effects	Suggests VEGFR2 contributes to MK differentiation in specific contexts; unclear relevance in ID.	Coppola et al. (2006)
VEGF/CXCR4	In vivo: BALB/c mice	VEGFR1 activation ➔ increased MK maturation (↑ CXCR4, MK migration to vascular niche, ↑ ploidy)	Defines mechanism linking angiogenic signaling to thrombocytosis; likely relevant in ID	Pitchford et al. (2012)
	In vivo: rat model on an ID diet	Anemia and thrombocytosis present ↑ MK number, vascularization, VEGF and CXCR4 expression	Direct evidence of angiogenic activation in ID‐associated thrombocytosis.	Garcia et al. (2021)
HIF‐2α/VEGF	In vitro: HEL and CMK cell lines and cord blood‐derived HSCs In vivo: rat model on an ID diet	ID ➔ ↑ HIF‐2α; ↑ VEGF; ↑MK differentiation and proplatelet formation	Supports hypoxia/iron‐sensing pathway driving megakaryopoiesis in ID	Jimenez et al. (2015)
TfR2	In vivo: A murine bone marrow chimera mice model with Tfr2 specifically deleted in the bone marrow compartment	TfR2 deletion ➔ ↑ erythropoiesis; ↑ EPO sensitivity	Defines a role for Tfr2 in regulating erythropoiesis in response to iron availability. This study does not directly address thrombocytosis.	Nai et al. (2015)
	In vitro: primary human MEPs In vivo: Tmprss6 KO mice and mice model on an ID diet.	ID or TFR2 KD ➔ ↓ ERK, ↓ proliferation, ↑ MK commitment	Direct mechanism: iron deficiency biases MEP fate toward MK lineage.	Xavier‐Ferrucio et al. (2019)

In this context, the present article aims to critically review the proposed mechanisms underlying thrombocytosis observed in iron‐deficiency anemia, addressing the main theoretical models and associated molecular pathways. By gathering and discussing these different perspectives, the study seeks to contribute to a more integrated understanding of the phenomenon and to foster the development of new hypotheses and investigative approaches in the field of hematology.

### 1.1 Vascular Endothelial Growth Factor (VEGF) Signaling in Megakaryocyte Maturation and Platelet Production

A robust body of evidence supports a key role for VEGF signaling in the regulation of megakaryocyte (MK) maturation and platelet production [17]. The distinct roles of VEGF receptors in megakaryopoiesis across experimental models are summarized in Table 2. Casella et al. investigated the expression dynamics of VEGF receptors during the unilineage differentiation of human CD34+ hematopoietic progenitor cells (HPCs) into MKs. Their work demonstrated that the expression of VEGF receptor 1 (Flt1 or VEGFR1) is upregulated during MK differentiation, with peak expression at intermediate differentiation stages [17]. Importantly, both VEGF and PIGF (Placental Growth Factor, a VEGFR1‐specific ligand) enhance MK polyploidization in vitro [17]. Inhibition of the VEGF‐VEGFR1 interaction, using either soluble VEGFR1 receptors or neutralizing antibodies, significantly impairs MK polyploidization, underscoring the functional importance of this pathway [17]. These findings suggest the existence of an autocrine and/or paracrine regulatory loop, whereby the activation of VEGFR1 promotes MK maturation.

**TABLE 2:** VEGF receptor functions in Megakaryopoiesis.

Model/system	VEGFR1 (Flt1)	VEGFR2 (KDR)	Key findings and summary	References
Primary human CD34+ cells	↑ VEGFR1 during MK differentiation VEGF/PlGF (VEGFR1 ligands) ➔ ↑ MK polyploidization VEGFR1 blockade ➔ ↓ MK maturation	Low in quiescent HPCs, downregulated early; re‐expressed during late stages of MK maturation Minor contribution to maturation	VEGFR1 is primary driver of MK maturation	[17]
In vivo mouse models	Activation (VEGF‐A/PlGF) ➔ ↑ MK ploidy; ↑ platelets; ↑ CXCR4; ↑ migration to vascular niche	VEGFR2 activation (VEGF‐E) ➔ no effect on platelets or MK maturation VEGFR2 blockade does not block VEGF‐A165 effect	VEGFR1 is sufficient for thrombocytosis and MK maturation/migration; VEGFR2 not required in vivo	[14]
Cell lines (TF1, K562, etc.)	Less commonly studied; endogenous Flt1 may be low or absent	VEGFR2 overexpression + VEGF stimulation ➔ ↑ MK markers expression, polyploidy, proliferation VEGFR2 inhibition ➔ ↓ MK maturation	VEGFR2 effects are context dependent and model‐specific	[17, 18]
Iron deficiency models	↑ VEGF/VEGFR1 signaling; ↑ MK number and ploidy; ↑ CXCR4	Not specifically addressed	VEGFR1 axis upregulation associated with reactive thrombocytosis in iron deficiency, independently of TPO	[4, 14, 17]

Extending these observations to an in vivo context, Pitchford et al. provide compelling evidence that VEGFR1 signaling increases MKs and platelets. Systemic administration of the VEGFR1‐selective agonist PlGF‐2 in a murine model robustly increased MK maturation (increased ploidy) in the bone marrow as well as circulating platelet counts. Thus, both in vitro and in vivo data converge to support a model in which VEGFR1 signaling promotes MK maturation and platelet formation, particularly in response to environmental cues such as hypoxia [14].

While the role of VEGFR1 in MK maturation is well documented, the involvement of VEGFR2 (KDR) appears to be more nuanced and context dependent. In the human TF1 cell line engineered to overexpress VEGFR2, VEGF stimulation promotes proliferation and survival while inducing megakaryocytic differentiation, characterized by increased expression of MK markers and polyploidization, both in the absence and presence of GM‐CSF. These effects were associated with G0/G1 cell cycle arrest. Inhibition of VEGFR2 signaling in primary MK‐differentiating cultures significantly reduced MK differentiation, suggesting that VEGFR2 can contribute to MK lineage commitment and maturation in specific cellular contexts [18].

However, these in vitro findings are not fully recapitulated in vivo. In murine models, selective activation of VEGFR2—either through combined administration of VEGF with VEGFR1 blockade or via VEGF‐E, a VEGFR2‐specific ligand—did not increase platelet counts or significantly alter megakaryocyte ploidy, maturation, or spatial distribution within the bone marrow [14].

Together, these observations suggest that although VEGFR2 can contribute to megakaryocytic differentiation under specific in vitro conditions, it is not sufficient to drive thrombocytosis in vivo. Instead, VEGFR1 appears to be the dominant receptor mediating VEGF‐induced megakaryopoiesis and platelet production.

### 1.2 Hypoxia Inducible Factors (HIFs), Iron Deficiency and VEGF Signaling

Hypoxia‐inducible factors (HIFs) function as key molecular regulators of cellular adaptation to hypoxia by controlling transcriptional responses to fluctuations in oxygen availability [19, 20, 21]. HIFs are heterodimeric transcription factors composed of an oxygen‐regulated α‐subunit, primarily HIF‐1α [22, 23], HIF‐2α [24] or HIF‐3α [25] and a constitutively expressed β‐subunit, ARNT (also known as HIF‐1β) [26].

The HIF‐1α/ARNT heterodimer uniquely governs the transcriptional activation of several glycolytic enzymes—including *PGK‐1*, *LDHA*, *PGM‐1*, and *PKM*—thereby positioning HIF‐1α as a key regulator of glycolytic flux under hypoxic conditions [20]. In contrast, HIF‐2α does not induce these glycolytic genes [20]. Nonetheless, both HIF‐1α and HIF‐2α regulate a subset of genes involved in broader hypoxic responses, such as *VEGF*, adrenomedullin (*ADM*), *N‐myc* downstream regulated gene 1 (*NDRG‐1*), and the glucose transporter *GLUT‐1*, indicating both shared and unique transcriptional programs [20]. HIFs play a role in hematopoietic stem cell maintenance, potentially mediated by HIF‐dependent VEGF signaling [19, 27, 28, 29].

HIF‐2α plays a role in the regulation of megakaryopoiesis, particularly under conditions of iron deficiency [13]. Studies have shown that ID enhances the megakaryocytic differentiation features in vitro in HEL and CMK cell lines, as well as in primary cord blood‐derived hematopoietic stem cells (CBHSC). Specifically, ID increases ploidy in HEL cells, upregulates megakaryocyte‐specific markers (CD41, CD61, CD42b) in CBHSC, and augments proplatelet formation in both HEL and CMK cell lines and CBHSC. Mechanistically, elevated HIF‐2α protein expression was observed in bone marrow megakaryocytes of iron‐deficient rats along with increased VEGF secretion in the culture supernatants of CBHSC grown under iron‐deficient conditions compared to iron‐replete conditions [13]. Additionally, supplementation of cultures with exogenous VEGF significantly increased the percentage of CD41‐positive megakaryocytic cells [13], thereby supporting a functional link between HIF‐2α, VEGF signaling, and megakaryocyte differentiation in the context of iron deficiency.

Under iron‐replete conditions, hypoxia‐induced stabilization of HIF‐2α in renal erythropoietin producing cells promotes erythropoiesis via induction of EPO [21, 22, 30, 31, 32, 33, 34]. However, when iron is limiting, the iron regulatory proteins (IRP1 and 2) can bind to the iron responsive element (IRE) in the 5′ untranslated region (UTR) of *HIF‐2α* mRNA, which leads to repression of HIF‐2α translation [21], reduces EPO synthesis and limits red blood cell production [21, 35]. Under these conditions, it is possible that persistent activation of the HIF‐2α/VEGF axis in the bone marrow may divert MEP differentiation toward the megakaryocytic lineage, contributing to the phenomenon of reactive thrombocytosis observed in iron deficiency [13]. This model suggests that HIF‐2α may mediate lineage bias at the level of the MEP, promoting erythropoiesis when iron is sufficient and favoring megakaryopoiesis via VEGF signaling when iron availability is low. Collectively, these data highlight a potential central role of HIF‐2α and iron status in orchestrating the balance between erythroid and megakaryocytic lineage commitment, revealing a metabolic control mechanism underlying hematopoietic fate decisions.

Corroborating these mechanistic findings, VEGF immunoreactivity increased in the bone marrow of iron‐deficient rats exhibiting reactive thrombocytosis. This increase in VEGF correlated with higher MK numbers and platelet counts, further implicating VEGF signaling in the augmentation of platelet production under pathological conditions, such as iron deficiency anemia [4].

### 1.3 VEGFR1–CXCR4 Axis and Megakaryocyte Migration

The chemokine receptor CXCR4 orchestrates MK positioning within the bone marrow and platelet output in response to angiogenic signaling. In in vivo investigations, stimulation of VEGFR1, achieved through administration of VEGF or the selective ligand PlGF‐2, resulted in a pronounced upregulation of CXCR4 on the surface of bone marrow MKs [14]. This molecular change was paralleled by a redistribution of MKs from the endosteal region, traditionally considered a site for immature or less active MKs, towards the sinusoidal vascular niche [14]. When this relocalization was inhibited by administration of the CXCR4 antagonist AMD3100, both the VEGFR1‐driven movement of MKs to the vascular niche and the associated increase in circulating platelet numbers (thrombocytosis) were inhibited [14]. These findings provide strong evidence that CXCR4‐mediated migration of mature MKs to the vascular niche is a necessary step for efficient platelet release in the reactive thrombocytosis that occurs in response to hypoxia or angiogenesis.

Complementary data further support the role of CXCR4 in thrombocytosis associated with ID. In a rat model of iron deficiency anemia, the authors observed a significant increase in MK numbers, and the MK has increased VEGF expression by immunohistochemistry. Increased CXCR4 expression was observed in what may have been immature MK in iron deficiency. Whether this was linked to MK positioning was not addressed, but is consistent with the data above [4].

### 1.4 TfR2 as a Modulator of Hematopoietic Lineage Commitment: Balancing Erythropoiesis and Megakaryopoiesis

TfR2 is a homolog of the classical Transferrin Receptor 1 (TfR1) but differs markedly in its tissue distribution and physiological function. Unlike the widely expressed TfR1, TfR2 is predominantly found in hepatocytes and hematopoietic progenitors as well as erythroid lineage cells, where it serves a regulatory role rather than facilitating iron transport. Unlike TFR1, which imports iron into cells, TfR2 functions as an iron sensor. The affinity of TfR2 for holo‐transferrin (holo‐TF) is lower than that of TfR1. This lower affinity may be functionally significant for iron sensing rather than iron uptake [36, 37, 38, 39]. Recent mechanistic insights support this sensory role: TfR2 shedding is directly inhibited by holo‐TF binding in a dose‐dependent manner. A mutant form of TfR2 unable to bind holo‐TF lacked this regulatory mechanism [37].

Loss‐of‐function mutations in *TFR2* are responsible for type 3 hereditary hemochromatosis, which is characterized by systemic iron overload resulting from inappropriately low levels of hepcidin consistent with a role for TFR2 in hepatocytes signaling when adequate iron is available [40, 41]. While the hepatic function of TFR2 as a sensor of circulating iron and a key regulator of hepcidin expression is well‐established [42, 43, 44], the role of TFR2 in the hematopoietic system, especially in the regulation of hematopoietic lineage commitment and differentiation of progenitors such as the Megakaryocyte‐Erythroid Progenitor (MEP) has only recently been explored.

Functional studies in mice demonstrate the physiological role of TfR2‐mediated iron sensing in erythropoiesis. Iron deficiency in control mice closely phenocopies the effects of erythroid‐specific Tfr2 deletion, including increased numbers of erythroblasts and reduced apoptosis within the erythroid compartment [45]. These findings suggest that both low iron availability or absence of Tfr2 leads to a compensatory increase in erythroid output. Importantly, this apparent expansion of erythroid progenitors reflects a compensatory response to impaired iron availability rather than effective erythropoiesis and does not translate into improved red blood cell production under iron‐deficient conditions. Integrating mechanistic and in vivo data, there is a proposed model in literature in which erythroid TfR2 acts as a sensor for iron availability—specifically, for circulating holo‐TF—and modulates erythrocyte production accordingly, likely by influencing the sensitivity of erythroid progenitors to EPO [36, 45]. This iron‐sensing mechanism appears to enable the erythroid compartment to adapt red blood cell production to systemic iron availability, thus preserving iron homeostasis and optimizing erythropoiesis [36].

Nevertheless, studies diverge on how TfR2 deletion or low iron affects erythroid output and hematopoietic lineage commitment, particularly in the balance between erythropoiesis and megakaryopoiesis. While Nai et al. concluded that Tfr2 acts as a limiting factor for RBC production [45], Xavier‐Ferrucio et al. reported that low iron conditions, whether induced genetically (Tmprss6−/− mice), by dietary iron deficiency, or by *TFR2* knockdown in human MEPs, bias lineage output toward the megakaryocytic lineage at the expense of erythroid and bipotent colonies in colony‐forming unit (CFU) assays [12]. Mechanistically, this Mk bias was associated with decreased MEP proliferation and reduced extracellular signal‐regulated kinase (ERK) phosphorylation under low iron or *TFR2* knockdown [12]. Therefore, reduced proliferation of MEP induced by reduced ERK activity itself may favor Mk commitment from MEPs, accounting for elevated platelet counts in iron deficiency anemia [46].

In line with these observations, another study demonstrated that megakaryocytic differentiation of the K562 cell line was accompanied by a marked reduction in TfR2 mRNA expression, indicating transcriptional downregulation of TfR2 during megakaryopoiesis [47]. Although this study did not establish a causal relationship between TfR2 downregulation and lineage commitment, it identified an association that is compatible with the findings reported by Xavier‐Ferrucio et al. [12].

The apparently contradictory findings are likely due to differences in the stage of hematopoiesis assessed, the integration of proliferation and differentiation signals, and the severity and duration of iron deficiency in the models used. Nai et al. primarily assessed erythropoiesis in vivo, focusing on mature erythrocyte output and late‐stage erythroid progenitor survival under conditions of mild and relatively short‐term iron deficiency. In this context, loss of Tfr2 function was associated with increased expression of EPO‐responsive genes and reduced apoptosis within the erythroid lineage, supporting enhanced survival and differentiation of erythroid‐committed progenitors [12, 45]. In contrast, Xavier‐Ferrucio et al. examined early hematopoietic fate decisions at the level of bipotent megakaryocyte–erythroid progenitors using in vitro assays under conditions of chronic and more severe iron limitation. In this setting, iron deficiency or TFR2 knockdown resulted in reduced MEP proliferation, diminished ERK phosphorylation, and a bias toward megakaryocytic colony formation at the expense of erythroid and bipotent colonies [12, 40].

In summary, TFR2 loss by shRNA mediated knockdown or in response to iron deficiency may promote megakaryocytic commitment at the progenitor level. Simultaneously, it may enhance EPO sensitivity in erythroid‐committed cells, supporting their survival and differentiation. These dual effects depend on the developmental stage examined and the severity of iron restriction [12, 45].

### 1.5 MAPK/ERK Pathway and Megakaryocyte Differentiation

Mechanistically, ERK activation leads to the phosphorylation of downstream targets, including members of the Ets family and AP‐1 transcription factors, as well as other kinases such as p90 ribosomal S6 kinase (RSK), which collectively modulate gene expression programs required for MK fate [16, 48, 49, 50, 51, 52].

The effects of ERK1/2 activation on cell proliferation and fate are highly dose and context‐dependent. Specifically, sustained and/or high‐level ERK1/2 activity—such as that induced by robust TPO stimulation in primary progenitor cells [53]—can promote cell cycle exit and differentiation, whereas transient ERK1/2 activation tends to promote proliferation [54, 55].

The MAPK/ERK pathway can regulate the cell cycle [56, 57, 58]. Reduced ERK phosphorylation may decrease cell cycle progression, which, in turn, could be associated with an increased propensity of MEPs to differentiate down the megakaryocytic rather than the erythroid lineage [12]. RNAseq of human CD34+ derived cells showed that cell cycle genes were among the most differentially expressed genes between MEP and MK progenitors and between MEP and erythroid progenitors, and also showed that the cell cycle of MK progenitors was decreased compared to erythroid progenitors [46].

Interestingly, the addition of VEGF to MEP also reduces ERK phosphorylation at the progenitor level [12], so both hypotheses converge to the same conclusion, that is, a slower cell cycle of MEP might drive MK commitment. Thus, it is plausible to hypothesize that the downregulation of MAPK/ERK pathway at the MEP level plays a role in driving this cell fate decision [12, 46], although the mechanisms involved still require further elucidation.

The phosphorylated VEGF receptor triggers multiple intracellular signaling pathways which can activate Protein Kinase C (PKC) and the Raf/MEK/ERK pathways [56, 57, 58]. It may be related to MK maturation, migration and proplatelet formation (PPF). Although MEK/ERK promotes erythroid over Mk fate specification at the MEP level, once cells are committed to the Mk lineage the activation of the MEK–ERK signaling cascade facilitates key processes during MK maturation, most notably polyploidization and the upregulation of late‐stage surface markers such as GPIb (CD42b) and GPIIb (CD41) [59, 60, 61, 62, 63, 64, 65]. Inhibition of MEK–ERK within developing megakaryocytes hampers maturation, resulting in a retention of immature markers like CD34 [59] and reduction in MK ploidy [66, 67, 68], underscoring the pathway's essential role in late‐stage MK differentiation [53, 59]. ERK1/2 activity is essential for the migration of mature murine BM‐derived MKs toward stromal cell‐derived factor 1α (SDF1α) gradients, and for PPF in fetal liver‐derived MKs [59]. ERK1/2 phosphorylation was observed upon adhesion to fibrinogen during PPF, suggesting a mechanistic link between ERK1/2 activation and cytoskeletal reorganization necessary for platelet release [59].

The role of the ERK1/2 pathway in human primary MK differentiation has not been fully elucidated. Whereas some investigations using human primary hematopoietic progenitors demonstrate enhanced polyploidization or negligible changes in differentiation markers upon MEK inhibition [53, 69], other studies report impaired or delayed MK maturation and polyploidization following ERK1/2 blockade [66, 68]. The discrepancies between these studies may be attributable to species differences (human vs. mouse), variations in the source of primary cells (cord blood vs. adult bone marrow or peripheral blood), and differences in culture conditions such as cytokine supplementation and serum concentration. The timing and dosage of MEK inhibitor application, as well as the pharmacological characteristics of the inhibitors themselves (e.g., PD98059 vs. U0126 or PD184161), are also likely to contribute to these divergent outcomes. The endpoints used to assess differentiation (e.g., surface marker expression vs. polyploidy profile or proplatelet formation) may capture different aspects of MK maturation, further complicating direct comparison across studies [48].

### 1.6 TET2, Iron and Erythroid/Megakaryocytic Differentiation

The ten‐eleven translocation methylcytosine dioxygenase 2 (TET2) functions as a key regulator of DNA hydroxymethylation in both mouse and human hematopoietic systems. TET2 is a member of the ten‐eleven translocation (TET) family of enzymes, which belong to the superfamily of Fe(II)‐ and 2‐oxoglutarate–dependent dioxygenases. TET2 catalyzes the oxidation of 5‐methylcytosine (5mC) to 5‐hydroxymethylcytosine (5hmC), a reaction integral to DNA demethylation and epigenetic regulation [70, 71]. Its optimal catalytic activity requires both 2‐oxoglutarate, an intermediate of the Krebs cycle, and ferrous iron (Fe^2+^), which binds within the catalytic center of the protein [72]. At this site, Fe^2+^ is required for the oxidation of 5‐methylcytosine (5 mC) in DNA, converting it into 5‐hydroxymethylcytosine and further oxidized derivatives [71, 73]. Experimental disruption of TET2, whether via genetic knockout, loss‐of‐function mutation, or RNA interference, consistently results in a significant global reduction of 5hmC levels in hematopoietic cells [74, 75, 76, 77].

The distribution of 5hmC is dynamically regulated during hematopoiesis, with the highest levels detected in primitive hematopoietic stem cells (HSCs) and multipotent progenitors (MPPs), and a progressive decline as differentiation proceeds [75, 76]. This specificity is further reinforced by the strong positive correlation between 5hmC abundance and both transcriptional activity and chromatin accessibility, as shown by 5hmC‐seq, ATAC‐seq, and RNA‐seq datasets correlation [75]. TET2 deficiency impairs erythroid and megakaryocytic differentiation [74, 75, 77]. However, given that TET2 is an iron‐dependent dioxygenase [70], an important unresolved question is whether iron deficiency may directly impair its enzymatic activity in hematopoietic progenitors.

This raises the hypothesis that reduced iron availability in MEPs may impair TET2 activity, leading to altered DNA hydroxymethylation patterns and contributing to lineage bias. Such epigenetic modulation could act in parallel with previously described signaling pathways, including reduced intracellular ERK phosphorylation under low iron conditions [12], further reinforcing megakaryocytic commitment.

This hypothesis could be experimentally tested by modulating intracellular iron availability in primary human hematopoietic stem and progenitor cells or sorted MEPs, followed by assessment of TET2 activity through quantification of 5‐hydroxymethylcytosine levels. Integration with RNA‐seq and chromatin accessibility assays could further define the downstream transcriptional consequences. In addition, rescue experiments using iron supplementation or ascorbate—known to enhance TET activity [72]—could help determine whether restoration of TET2 function reverses lineage bias under iron‐deficient conditions.

Conflicting findings have been reported regarding the effect of Tet2 loss on MK ploidy. One study reports a reduction in the proportion of hyperploid (> 32 N) megakaryocytes and a concomitant increase in lower ploidy MKs in Tet2‐deficient mice [77]. In contrast, another publication describes that loss of Tet2, whether by pan‐hematopoietic, megakaryocyte‐specific, or clonal hematopoiesis (CHIP) modeling, results in an increased number of larger MKs with higher ploidy (16–32 N) [78].

The discrepancies between the studies regarding the effects of Tet2 loss on megakaryocyte ploidy may be attributed to several key factors. First, they use distinct models: the first one employs a full germline *Tet2* knockout, while the second uses conditional and CHIP models that better mimic age‐related, somatic mutations. Second, age plays a role: the first study assessed mice in the context of aging and CHIP, while the second one did not control for age. Third, their definitions of “higher ploidy” differ, with the first paper focusing on > 32 N MKs and the second on the 16–32 N range, which may contribute to non‐overlapping results. Finally, methodological differences such as flow cytometry versus immunofluorescence and varying gating strategies may further explain the contrasting findings [77, 78].

### 1.7 Toward an Integrated Mechanistic Framework of Thrombocytosis in Iron Deficiency

Although these mechanisms have traditionally been investigated in isolation, accumulating evidence suggests that they represent interconnected components of a coordinated response to iron deficiency [9]. In this context, iron deficiency can be conceptualized as a central metabolic constraint that simultaneously influences multiple regulatory layers of hematopoiesis. At the progenitor level, reduced iron availability may be sensed through pathways involving transferrin receptor 2 (TfR2), leading to decreased ERK signaling, impaired proliferation of megakaryocyte–erythroid progenitors (MEPs), and a bias toward megakaryocytic differentiation [12]. In parallel, activation of HIF‐2α under iron‐deficient conditions promotes VEGF production, which in turn enhances megakaryocyte maturation and platelet release through VEGFR1 signaling and CXCR4‐mediated migration to the vascular niche [13, 14, 17]. Additionally, iron‐dependent epigenetic regulators such as TET2 may further modulate lineage commitment through effects on DNA hydroxymethylation [76], although this remains to be fully elucidated.

**FIGURE 1:**
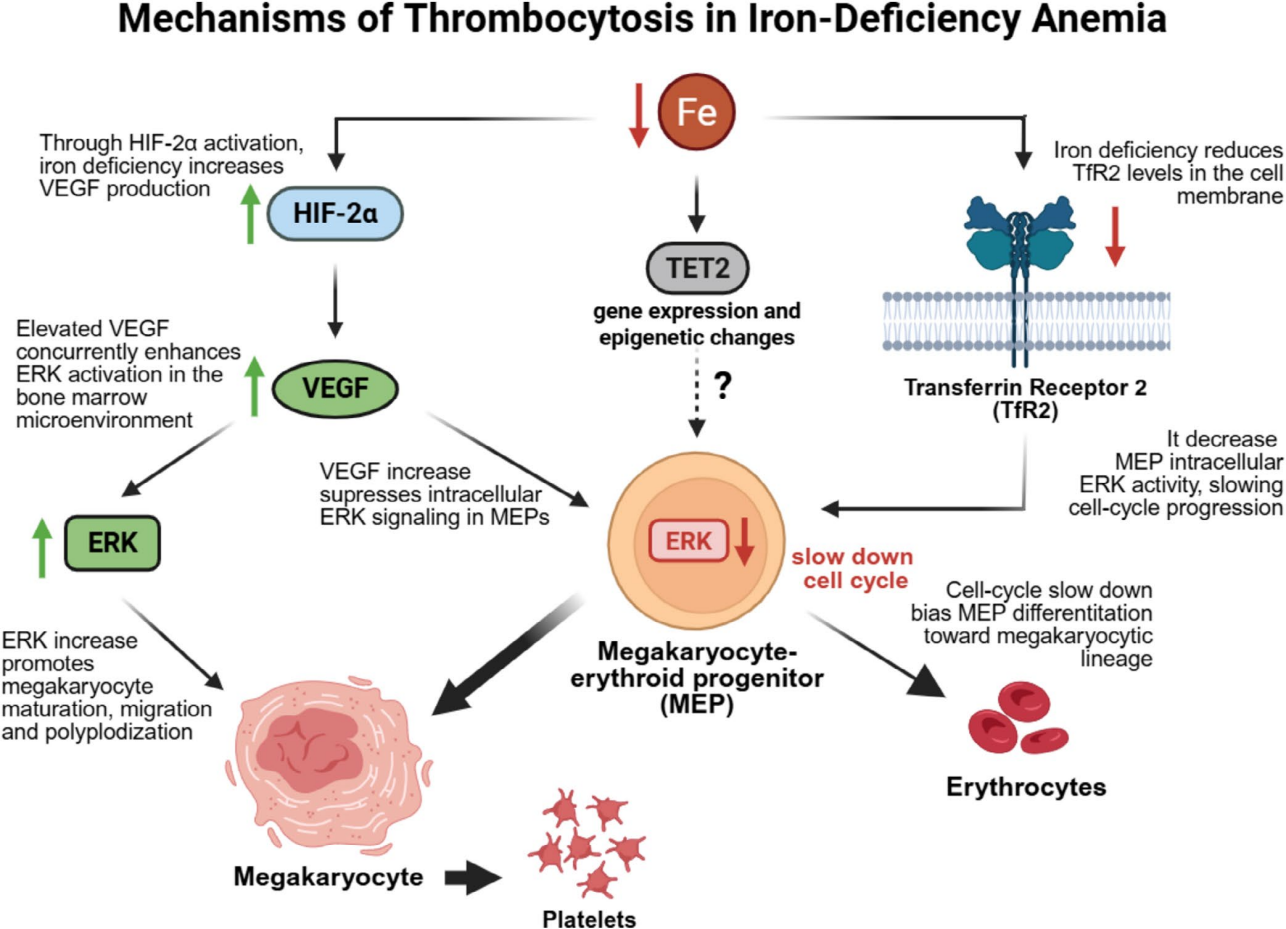
Integrated model of thrombocytosis in iron‐deficiency anemia. Iron deficiency acts as a central metabolic constraint that affects multiple interconnected pathways regulating hematopoietic fate. Reduced iron availability leads to decreased transferrin receptor 2 (TfR2) signaling, resulting in reduced ERK activation in megakaryocyte–erythroid progenitors (MEPs), impaired proliferation, and a bias toward megakaryocytic differentiation. In parallel, activation of HIF‐2α under iron‐deficient conditions promotes VEGF production, which exerts context‐dependent effects: enhancing ERK signaling within the bone marrow microenvironment while suppressing intracellular ERK activity in MEPs. This dual regulation contributes to megakaryocyte maturation, migration, and polyploidization. Additionally, iron‐dependent epigenetic regulators such as TET2 may further modulate gene expression and lineage commitment, although their precise role remains to be fully elucidated. Together, these pathways converge at the level of the MEP to promote megakaryocyte differentiation and increased platelet production, ultimately resulting in thrombocytosis.

Together, these pathways support a hierarchical and interconnected model in which iron availability regulates hematopoietic fate decisions through coordinated effects on signaling, metabolism, and epigenetic regulation, ultimately contributing to thrombocytosis in iron‐deficiency anemia (Figure 1).

## 2 Conclusion

Thrombocytosis in the setting of iron deficiency anemia remains an area with substantial gaps in understanding and continues to attract significant interest. Current evidence is heterogeneous, and the hypotheses discussed in this review still require validation to fully elucidate the mechanisms driving the increased platelet counts observed in this context.

While a lineage bias at the megakaryocyte–erythroid progenitor level is a leading hypothesis, the precise cascade of molecular events involved in this fate decision is likely far more complex and interconnected. Multiple plausible mechanisms have been proposed, each contributing a fragment of the overall picture. Activation of the HIF‐2α/VEGF pathway emerges as a strong candidate in promoting megakaryocyte maturation and migration; however, its role as the primary initiator of lineage skewing remains to be firmly established. Likewise, dysregulation of TfR2 signaling and the MAPK/ERK pathway has been implicated in modulating MEP commitment, though conflicting data across studies preclude definitive conclusions. Moreover, the impact of iron deficiency on essential iron‐dependent epigenetic regulators, such as TET2, remains largely unexplored and highly speculative.

Beyond its mechanistic relevance, understanding the pathways linking iron deficiency to thrombocytosis may have important clinical implications. While current management primarily relies on iron repletion [79], a more detailed understanding of these mechanisms raises the possibility of targeted therapeutic approaches in selected cases of severe or persistent thrombocytosis. Modulation of VEGF signaling or interference with the VEGFR1–CXCR4 axis could theoretically reduce megakaryocyte maturation, migration, and platelet production [14, 17]. Additionally, targeting intracellular signaling pathways that regulate progenitor cell fate, such as the MAPK/ERK pathway, may represent a strategy to influence lineage commitment [12]. However, these approaches remain speculative and would require careful evaluation of safety, given the central role of these pathways in hematopoiesis and vascular biology. Future studies are needed to determine whether modulation of these pathways can be safely translated into clinical practice, particularly in patients with extreme thrombocytosis or increased thrombotic risk.

## Funding

This work was supported by the National Council for Scientific and Technological Development (CNPq; grants #306882/2023‐0 and #177757/2024‐8 to STOS) and the São Paulo Research Foundation (FAPESP; grant #2025/05991‐2 to STOS). JVF is supported by a MD–PhD scholarship from the São Paulo Research Foundation (FAPESP; grant #2024/11135‐9).

## Ethics Statement

Ethical approval was not required for this study, as it is a review article based on previously published literature.

## Consent

The authors have nothing to report.

## Conflicts of Interest

The authors declare no conflicts of interest.

## Data Availability

Data sharing not applicable to this article as no datasets were generated or analysed during the current study.
